# Schistosomiasis was not associated with higher HIV-1 plasma or genital set point viral loads among HIV seroconverters from four cohort studies

**DOI:** 10.1371/journal.pntd.0007886

**Published:** 2019-11-20

**Authors:** Aaron F. Bochner, W. Evan Secor, Jared M. Baeten, Govert J. van Dam, Adam A. Szpiro, Sammy M. Njenga, Paul L. A. M. Corstjens, Romel D. Mackelprang, Nelly R. Mugo, Julie Overbaugh, Connie Celum, Andrew Mujugira, R. Scott McClelland, Ruanne V. Barnabas

**Affiliations:** 1 Department of Epidemiology, University of Washington, Seattle, Washington, United States of America; 2 Department of Global Health, University of Washington, Seattle, Washington, United States of America; 3 Division of Parasitic Diseases and Malaria, Center for Global Health, Centers for Disease Control and Prevention, Atlanta, Georgia, United States of America; 4 School of Medicine, University of Washington, Seattle, Washington, United States of America; 5 Department of Parasitology, Leiden University Medical Center, Leiden, the Netherlands; 6 Department of Biostatistics, University of Washington, Seattle, Washington, United States of America; 7 Kenya Medical Research Institute, Nairobi, Kenya; 8 Department of Cell and Chemical Biology, Leiden University Medical Center, Leiden, the Netherlands; 9 Division of Human Biology, Fred Hutchinson Cancer Research Center, Seattle, Washington, United States of America; 10 Infectious Diseases Institute, College of Health Sciences, Makerere University, Kampala, Uganda; PUCRS, BRAZIL

## Abstract

**Background:**

Many regions of sub-Saharan Africa experience a high prevalence of both schistosomiasis and HIV-1, leading to frequent coinfection. Higher plasma HIV-1 viral loads are associated with faster disease progression and genital HIV-1 loads are a primary determinant of HIV-1 transmission risk. We hypothesized that schistosome infection would be associated with higher HIV-1 viral loads in plasma and genital samples.

**Methods/Principal findings:**

We utilized data from individuals who HIV-1 seroconverted while enrolled in one of four prospective cohort studies. Plasma and genital viral loads collected 4–24 months after the estimated date of HIV-1 acquisition, but prior to antiretroviral therapy initiation, were included. Detection of circulating anodic antigen in archived blood samples, collected prior to HIV-1 seroconversion, identified participants with active schistosomiasis; immunoblots determined the schistosome species causing infection. Our analysis included 370 HIV-1 seroconverters with plasma viral load results, of whom 82 (22%) had schistosomiasis. We did not find a statistically significant association between schistosomiasis and higher HIV-1 set point plasma viral loads (-0.17 log_10_ copies/ml, 95% CI -0.38 to 0.03); *S*. *mansoni* infection was associated with a lower set point (-0.34 log_10_ copies/ml, 95% CI -0.58 to -0.09). We found no association between schistosomiasis and cervical (+0.07 log_10_ copies/swab, 95% CI -0.20 to 0.34) or vaginal (+0.11 log_10_ copies/swab, 95% CI -0.17 to 0.39) set point viral loads; *S*. *haematobium* infection was associated with lower cervical viral loads (-0.59 log_10_ copies/swab, 95% CI -1.11 to -0.06).

**Conclusions/Significance:**

These results do not support the hypotheses that schistosome coinfection increases plasma or genital HIV-1 viral loads.

## Author summary

Schistosomiasis is a parasitic disease that is common in many parts of sub-Saharan Africa most affected by the HIV-1 epidemic. Schistosomiasis causes genital damage when schistosome ova become lodged in the female genital tract, inducing inflammation that may elevate HIV-1 genital viral loads and increase the risk of HIV-1 transmission. Schistosomiasis may also promote viral replication by facilitating cell-to-cell transmission of HIV-1, elevating HIV-1 plasma viral load levels. Using data from 370 individuals residing in Kenya or Uganda who acquired HIV-1 while participating in one of four prospective cohort studies, we tested the hypotheses that schistosomiasis increases plasma and genital viral load levels. We found no evidence that individuals with schistosomiasis had higher set point plasma viral load levels, a measure of viral replication obtained during the set point period 4–24 months after HIV-1 acquisition when viral load levels remain relatively stable. Additionally, we found no evidence that schistosomiasis was associated with higher female set point genital viral loads measured from vaginal or cervical swabs. Unexpectedly, we found that *S*. *mansoni* infection was associated with a decline in plasma viral load levels while *S*. *haematobium* infection was associated with a decline in cervical viral load levels. Thus, our results do not support the hypotheses that schistosomiasis increases plasma and genital HIV-1 viral loads.

## Introduction

Schistosomiasis is a parasitic disease affecting approximately 200 million individuals globally [1]. With 90% of schistosome infections occurring in sub-Saharan Africa [2, 3], there is significant geographical overlap between areas with high schistosomiasis and HIV-1 prevalence [4]. Other HIV-1 coinfections have been found to increase HIV-1 transmission risk or disease progression [5–7], and these facts led to the hypothesis that schistosome coinfection plays a role in driving the HIV-1 epidemic in sub-Saharan Africa.

Some evidence has suggested that schistosome infection increases the rate of systemic and genital HIV-1 viral replication, potentially resulting in accelerated rates of disease progression and increased transmission risk [8]. A recent study in Tanzania found higher set point plasma viral load levels among individuals with schistosomiasis [9], and schistosome-infected macaques had higher viral loads after SHIV acquisition [10, 11]. One mechanism proposed to explain these associations is that individuals with schistosomiasis have denser concentrations of HIV co-receptors CCR5 and CXCR4 on CD4+ T-cells, which could ease cell-to-cell spread of HIV-1, promoting viral replication and disease progression [12]. Additionally, individuals with *S*. *mansoni* infection had increased expression of α4β7 on blood CD4+ T cells [13], and increased pre-HIV infection expression of α4β7 has been associated with higher set-point plasma viral loads [14]. Lastly, female schistosomes lay hundreds of eggs per day that can become deposited in host female genital organs, triggering an inflammatory response with recruitment of leukocytes to the genital epithelium [15–17]. Higher concentrations of leukocytes in the genital epithelium of women have been associated with increased HIV-1 genital viral loads [18]. Through this mechanism, schistosome coinfection has been hypothesized to increase HIV-1 genital viral loads [15, 19, 20], a primary determinant of HIV-1 transmission risk [21].

Using data obtained from individuals in Kenya and Uganda who acquired HIV-1, our objective was to evaluate if schistosome infections were associated with HIV-1 viral loads during the set point period 4–24 months after HIV-1 acquisition, when both plasma and genital HIV-1 viral load levels are relatively stable [22]. Two schistosome species cause schistosomiasis in the study area: *S*. *haematobium* and *S*. *mansoni* [3]. We hypothesized that individuals infected with either species would have higher viral loads in plasma samples. Because the two species have differing pathologies, with *S*. *haematobium* eggs more often found in the female genital tract [23, 24], we hypothesized a positive association between *S*. *haematobium* infection and genital viral loads, with an attenuated positive association among individuals infected by *S*. *mansoni*.

## Methods

### Human subjects research

All study protocols, which included provisions for future analyses of HIV-1 transmission risk factors, were approved by the University of Washington Human Subjects Division as well as ethics review committees at each study site. The study was also reviewed by the U.S. Centers for Disease Control and Prevention (CDC), which deemed CDC personnel to be non-engaged, as they had no contact with study participants or access to personal identifiers. All study participants were adults or emancipated minors under Kenyan and Uganda law and provided written informed consent. Additionally, this study was conducted with approval from the Kenyatta National Hospital—University of Nairobi Ethics and Research Committee (KNH-UON ERC), which requires that we release data from Kenyan studies (including de-identified data) only with their written approval for additional analyses.

### Study population

We utilized data obtained from individuals who experienced HIV-1 seroconversion while enrolled in one of four prospective cohort studies conducted in Kenya and Uganda. Three of these cohorts enrolled heterosexual HIV-1 serodiscordant couples: the Partners in Prevention HSV/HIV Transmission Study [25], the Couples Observational Study [26], and the Partners PrEP Study [27]. In total, these three cohorts followed more than 8,500 couples for 12 to 36 months, and 297 individuals seroconverted during follow-up. The fourth study was the Mombasa Cohort, an open cohort of female sex workers (FSW) that began enrollment in 1993. Through 2014, 3,471 women had enrolled and 332 had experienced HIV-1 seroconversion.

Individuals who HIV-1 seroconverted during study follow-up and who had a blood sample collected prior to seroconversion available for schistosomiasis testing were eligible for the present analysis. In addition, for the Partners in Prevention HSV/HIV Transmission Study and Couples Observational Study, we only included participants enrolled at study sites in Kenya and Uganda, where schistosomiasis is endemic [3]. Screening or mass-treatment for schistosomiasis were not included in the study protocol for any of the four cohorts. All HIV-1 seroconverters in the four cohorts were invited to continue their cohort enrollment. Seroconverters in the serodiscordant couples cohorts were invited to attend quarterly study visits, while seroconverters in the Mombasa Cohort were invited to monthly visits; at these visits, a blood sample was collected to measure plasma viral loads. Additionally, women in the Mombasa Cohort had vaginal and cervical swabs collected to measure genital viral loads. Vaginal swab collection was performed by rolling a Dacron swab for three full turns against the lateral vaginal wall, while cervical swabs were inserted into the cervical os and rotated two full turns (study procedures described previously [28]).

### Schistosomiasis and viral load testing

Schistosome infections were identified via a three-stage testing algorithm. Samples collected prior to HIV-1 seroconversion were initially tested by soluble egg antigen (SEA) ELISA to detect antischistosomal antibodies [29]. Since antibodies persist beyond the period of active infection, SEA ELISA-positive serum samples were tested for circulating anodic antigen (CAA) using the SCAA20 assay (detection threshold of 10 pg/ml) [30]; CAA becomes undetectable within two weeks after successful treatment with praziquantel [31]. To identify the schistosome species causing infection, CAA-positive samples had species-specific immunoblots performed for *S*. *mansoni* and *S*. *haematobium* [32, 33]. SEA ELISAs and species-specific immunoblots were performed by the Parasitic Diseases Branch of the CDC and CAA testing was performed at Leiden University Medical Center.

The assay used to measure plasma HIV-1 RNA levels varied across studies (Partners HSV/HIV: COBAS AmpliPrep/COBAS TaqMan HIV-1 RNA assay, version 1.0 [Roche Diagnostics]; Couples Observational Study and Partners PrEP Study: Abbott m2000 Real-Time HIV-1 RNA assay [Abbott]; Mombasa Cohort: Gen-Probe HIV-1 viral load assay [Gen-Probe Incorporated]). Genital swabs collected from the Mombasa Cohort were eluted in 1 ml of buffer, and the concentration of HIV-1 RNA was reported as HIV-1 copies/swab. Quantification of genital HIV-1 RNA was conducted using the Gen-Probe HIV-1 viral load assay (Gen-Probe Incorporated).

### Date of HIV-1 infection and HIV-1 set point

We estimated the date of HIV-1 acquisition using both serology and plasma viral load results. Study participants were tested for HIV-1 seroconversion at all routine study visits (monthly or quarterly). For the serodiscordant couples cohorts, dual rapid HIV-1 antibody tests were performed, followed by a confirmatory HIV-1 enzyme immunoassay and Western blot. For the Mombasa Cohort, HIV-1 ELISA testing was performed, with positive results confirmed by a second ELISA. In all cohorts, once a positive serology was confirmed, HIV-1 RNA levels were measured in plasma samples collected at the seroconversion visit and preceding visits until a visit prior to HIV-1 acquisition was identified.

For individuals with detectable HIV-1 RNA prior to HIV-1 seroconversion, we estimated the date of HIV-1 acquisition to be 17 days prior to the date of the first positive HIV-1 RNA result. For those whose first detectable HIV-1 RNA occurred at the seroconversion visit, the date of HIV-1 infection was estimated to be at the midpoint between the last seronegative and first seropositive visit. For this study we excluded participants without detectable plasma HIV-1 RNA prior to seroconversion who had >1 year between their last seronegative and first seropositive visit, since those individuals’ date of HIV-1 acquisition could not be estimated with adequate precision.

We defined the set point period as being between 4–24 months after HIV-1 infection. Thus, we included all eligible plasma or genital viral load samples collected 4–24 months after the estimated date of HIV-1 acquisition. We excluded any samples collected after the participant reported initiating antiretroviral therapy (ART); information on ART use was collected at all study visits. This approach is consistent with previous analyses conducted with these cohorts [34, 35].

### Statistical methods

We log_10_-transformed plasma and genital HIV-1 RNA concentrations to approximate a normal distribution. Samples with undetectable RNA levels were set equal to half the lower limit of quantification (120 copies/ml for plasma and 25 copies/swab for genital samples). Linear mixed-effects models with a random intercept for each individual evaluated if schistosomiasis was associated with plasma or genital set point viral load levels. Multivariable models evaluating set point plasma viral loads adjusted for a set of *a priori* confounders identified through a review of the literature: age [36], sex [37, 38], study, and four year bands of calendar year (Mombasa Cohort only, to account for time trends in HIV-1 transmission in that community) [36]. Multivariable models for genital viral loads included this same set of confounders with additional adjustment for plasma viral load levels obtained at the same study visit as genital viral loads. At the request of a reviewer, we also evaluated if hormonal contraceptives or sexually transmitted infections confounded the association between schistosomiasis and plasma or genital viral loads, and we found that their inclusion did not meaningfully change effect estimates (change of ≤0.02 log_10_ copies/ml).

We used analogous statistical models with the same sets of covariates to perform subgroup analyses by schistosome species. We included indicator variables for *S*. *mansoni*, *S*. *haematobium*, and infections caused by an undetermined species (CAA ≥10 pg/ml but negative immunoblot results for both species) into a single statistical model, with uninfected individuals (CAA negative results) as the reference group. For plasma viral loads, where we had more statistical power, we also performed exploratory subgroup analyses by sex, study population, and schistosome infection intensity levels, using CAA levels to classify infections as low (10–99 pg/ml), medium (100–999 pg/ml), or high burden (≥1000 pg/ml) since CAA levels correlate with worm burden [30]. Additionally, to address the possibility that schistosome infection status changed over time, we performed a sensitivity analysis, restricting the selection criteria to include only samples collected 4–12 months after HIV-1 infection. All analyses were conducted using Stata version 13.1 (Stata Corporation, College Station, TX).

## Results

### Descriptive statistics of the study population

**Fig 1:**
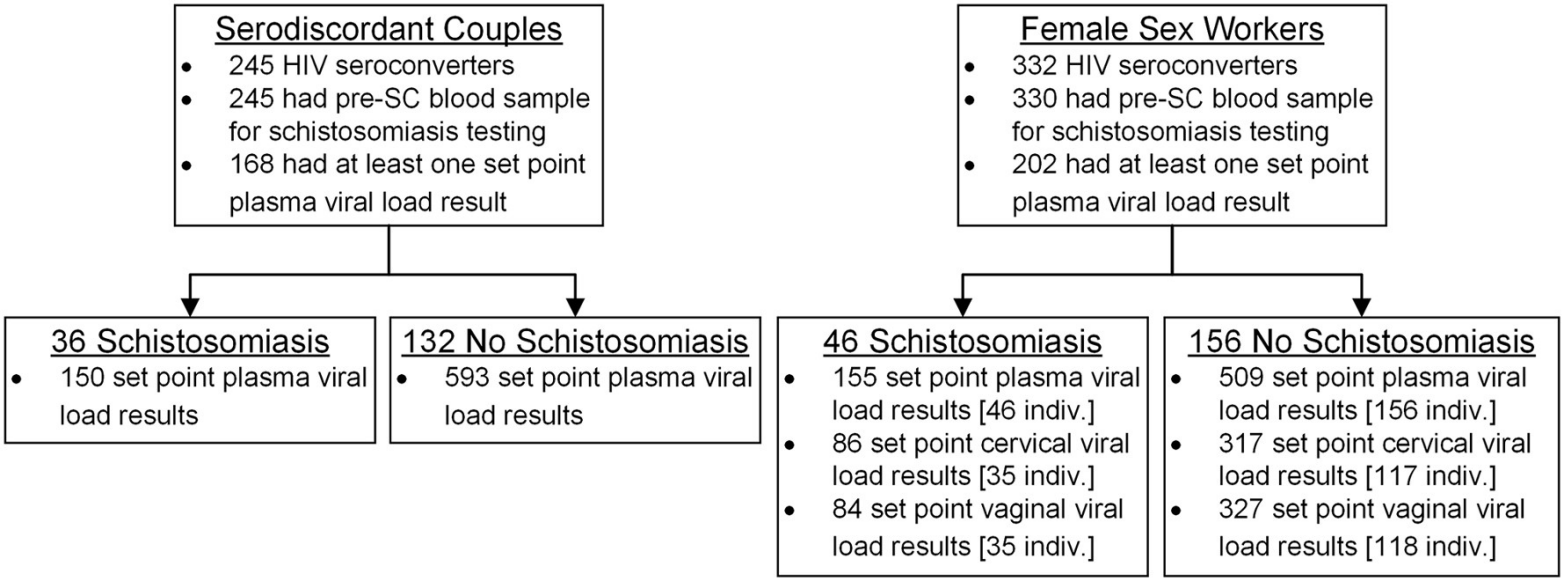
Eligibility and availability of samples from HIV-1 seroconverters in the serodiscordant couples and female sex worker cohorts.

A total of 245 Kenyan and Ugandan participants from the three serodiscordant couples cohorts and 332 FSWs from the Mombasa Cohort experienced HIV-1 seroconversion, and all but two individuals had a pre-seroconversion blood sample available for schistosomiasis testing (Fig 1). From these 575 individuals, 370 (64%) had at least one plasma viral load result from the set point period obtained prior to ART initiation (Table 1). In the serodiscordant couples cohorts 21% (36/168) of HIV-1 seroconverters had schistosomiasis, compared to 23% (46/202) among members of the Mombasa Cohort. The median number of days from collection of the sample used for schistosomiasis testing to the estimated date of HIV-1 acquisition was 14 (interquartile range [IQR], -17 to 46) for the serodiscordant couples cohorts and 74 (IQR, 47 to 127) for the Mombasa Cohort; negative numbers indicate that the participant acquired HIV-1 prior to the date of schistosomiasis testing. The median number of eligible set point viral load results per individual was 4 (IQR, 3 to 6) in the serodiscordant couples cohorts and 3 (IQR, 2 to 5) in the Mombasa Cohort.

**Table 1:** Characteristics of participants with eligible set point viral load results after HIV-1 seroconversion.

	Serodiscordant Couples HIV-1 Seroconverters		Female Sex Workers HIV-1 Seroconverters	
	Schistosome infected (N = 36)	Schistosome uninfected (N = 132)	Schistosome infected (N = 46)	Schistosome uninfected (N = 156)
Age at HIV-1 seroconversion				
16–24	5 (14%)	29 (22%)	10 (22%)	34 (22%)
25–34	16 (44%)	63 (48%)	25 (54%)	85 (54%)
≥35	15 (42%)	40 (30%)	11 (24%)	37 (24%)
Sex				
Female	15 (42%)	70 (53%)	46 (100%)	156 (100%)
Male	21 (58%)	62 (47%)	—	—
Education^1^				
<9 years	28 (78%)	88 (67%)	34 (74%)	98 (63%)
≥9 years	8 (22%)	44 (33%)	12 (26%)	58 (37%)
Married^2^				
Yes	35 (97%)	127 (96%)	28 (61%)	83 (53%)
No	1 (3%)	5 (4%)	18 (39%)	73 (47%)
Enrollment location				
Kenya	11 (31%)	63 (48%)	46 (100%)	156 (100%)
Uganda	25 (69%)	69 (52%)	—	—
Any unprotected sex^3^				
Yes	22 (61%)	95 (72%)	21 (46%)	88 (56%)
No	14 (39%)	37 (28%)	25 (54%)	68 (44%)
Number of sex partners^3^				
≤1	32 (91%)	120 (93%)	38 (83%)	118 (76%)
>1	3 (9%)	9 (7%)	8 (17%)	38 (24%)
Sexually transmitted infections^4^				
Yes	7 (19%)	15 (11%)	10 (22%)	27 (18%)
No	29 (81%)	117 (89%)	35 (78%)	127 (82%)
Contraceptive use, females only				
None	11 (73%)	43 (61%)	27 (59%)	70 (45%)
IUD/surgical	2 (13%)	1 (1%)	3 (7%)	2 (1%)
Implant/injectable	2 (13%)	22 (31%)	13 (28%)	55 (35%)
Oral contraceptive	0 (0%)	4 (6%)	3 (7%)	29 (19%)
Serodiscordant couples cohort				
Partners HSV/HIV Transmission Study	21 (58%)	67 (51%)	—	—
Couples Observational Study	1 (3%)	9 (7%)	—	—
Partners PrEP Study	14 (39%)	56 (42%)	—	—
Number of plasma viral load results per participant [Median (IQR)]	4 (3–6)		3 (2–5)	
Number of cervical viral load results per participant [Median (IQR)]	—	—	2 (1–4)	
Number of vaginal viral load results per participant [Median (IQR)]	—	—	2 (1–4)	
CAA levels, pg/ml [Median (IQR)]	495 (93–3264)	—	433 (56–2324)	—

^1^ Years of education at time of cohort enrollment.

^2^ For the serodiscordant couples cohorts, marital status at the time of study enrollment was assessed. For the FSW cohort, marital status at enrollment was categorized as ever married vs. never married because few participants were married.

^3^ For the serodiscordant couples cohorts, sexual behaviors were assessed over the prior month. Some individuals had missing values for number of sexual partners (n = 4). For the FSW cohorts, average sexual behaviors were calculated for each year of cohort follow-up.

^4^ For the serodiscordant couples cohorts, sexually transmitted infection testing (trichomoniasis, gonorrhea, and chlamydia) was done at enrollment. For the FSW cohort, sexually transmitted infection testing (trichomoniasis and gonorrhea) occurred at each study visit and was time-varying, and a few individuals lacked test results (n = 3).

### Schistosomiasis and plasma viral load

In total, there were 1,102 set point plasma viral load results from 288 HIV-1 seroconverters without schistosomiasis (mean = 4.50 log_10_ copies/ml) and 305 results from 82 seroconverters with schistosomiasis (mean = 4.32 log_10_ copies/ml). After controlling for age, sex, cohort, and year of HIV-1 acquisition, we found that HIV-1 seroconverters with schistosomiasis had set point plasma viral loads that were 0.17 log_10_ copies/ml lower (95% CI -0.38 to 0.03) than seroconverters without schistosomiasis (Table 2). To address the possibility that schistosomiasis status may have changed over time, we performed a sensitivity analysis including only samples collected 4–12 months after the estimated date of HIV-1 acquisition. In this sensitivity analysis, we obtained a point estimate of similar magnitude that achieved statistical significance (-0.22 log_10_ copies/ml, 95% CI -0.43 to -0.01). Additionally, we performed subanalyses by schistosome species (Table 2). Compared to individuals without schistosomiasis, individuals infected by *S*. *mansoni* had statistically significantly lower set point plasma viral loads (-0.34 log_10_ copies/ml, 95% CI -0.58 to -0.09). In contrast, individuals infected by *S*. *haematobium* had higher plasma viral loads compared to uninfected individuals (+0.33 log_10_ copies/ml, 95% CI -0.07 to 0.73), though the result did not achieve statistical significance.

**Table 2:** Associations between schistosomiasis and HIV-1 set point plasma viral load.

			Bivariate				Adjusted model^1^		
	**n obs**.	**(N indiv.)**	**Log**_**10**_ **copies/ml**^2^	**β**	**95% CI**	***P***	**β**	**95% CI**	***P***
No schistosomiasis	1102	(288)	4.50	Ref	—	—	Ref	—	—
Schistosomiasis^3^	305	(82)	4.32	-0.14	-0.34, 0.07	0.184	-0.17	-0.38, 0.03	0.094
	**n obs**.^4^	**(N indiv.)**	**Log**_**10**_ **copies/ml**^2^	**β**	**95% CI**	***P***	**β**	**95% CI**	***P***
No schistosomiasis	1102	(288)	4.50	Ref	—	—	Ref	—	—
*S*. *mansoni*	205	(53)	4.24	-0.30	-0.55, -0.06	0.015	-0.34	-0.58, -0.09	0.007
*S*. *haematobium*	62	(18)	4.61	0.41	0.01, 0.81	0.046	0.33	-0.07, 0.73	0.108
Undetermined species^5^	68	(18)	4.38	-0.05	-0.44, 0.35	0.818	-0.01	-0.40, 0.39	0.971

^1^ Adjusted for age (16-24/25-34/≥35), sex, and cohort, plus year of HIV-1 acquisition for the Mombasa Cohort (4-year bands).

^2^ The mean log_10_ copies/ml plasma viral loads for individuals with and without schistosomiasis, it does not take into account multiple observations per individual.

^3^ Individuals with antischistosomal antibodies (anti-SEA) and schistosome antigens (CAA >10 pg/ml).

^4^ There were 8 individuals (31 observations) who tested positive for both *S*. *mansoni* and *S*. *haematobium*.

^5^ These individuals had antischistosomal antibodies (anti-SEA) and schistosome antigens (CAA >10 pg/ml), but tested negative for both *S*. *mansoni* and *S*. *haematobium*.

Although we did not anticipate that schistosome infection intensity would impact plasma HIV viral load levels, we conducted an exploratory analysis and found that individuals with high intensity infections (CAA ≥1000 pg/ml) had lower set point plasma viral load levels (-0.44 log_10_ copies/ml, 95% CI -0.75 to -0.13) compared to those without schistosomiasis (S1 Table). We also performed exploratory subanalyses by sex and study population (S2 Table). We found that women with schistosomiasis had a near-significant association with lower set point plasma viral load levels (-0.23 log_10_ copies/ml, 95% CI -0.46 to 0.00) while among men with schistosomiasis we did not observe a strong association (0.04 log_10_ copies/ml, 95% CI -0.38 to 0.46). Additionally, we found that FSWs in the Mombasa Cohort with schistosomiasis had lower set point plasma viral load levels (-0.29 log_10_ copies/ml, 95% CI -0.55 to -0.02) compared to those without schistosomiasis (S2 Table).

### Schistosomiasis and genital viral load

Genital viral load results were only available for the Mombasa Cohort. Out of 202 women with eligible plasma viral load results, 152 had at least one eligible cervical viral load result and 153 had at least one eligible vaginal viral load result. The median number of cervical and vaginal viral load results per individual was 2 (IQR, 1 to 4).

We found that individuals with and without schistosomiasis had similar set point genital viral loads (Table 3). Individuals with schistosomiasis had cervical set point viral loads that were on average 0.07 log_10_ copies/swab (95% CI -0.20 to 0.34) higher than uninfected individuals, adjusting for age, year of HIV-1 acquisition, and plasma viral load. Adjusting for the same covariates, individuals with schistosomiasis had vaginal set point viral loads that were 0.11 log_10_ copies/swab (95% CI -0.17 to 0.39) higher than uninfected individuals. In a sensitivity analysis including only samples collected 4–12 months after the estimated date of HIV-1 acquisition, we still found no evidence that cervical (-0.02 log_10_ copies/ml, 95% CI -0.35 to 0.30) or vaginal (-0.14 log_10_ copies/ml, 95% CI -0.49 to 0.21) viral loads were associated with schistosome infection.

**Table 3:** Associations between schistosomiasis and HIV-1 genital viral loads in the Mombasa Cohort.

			Bivariate				Adjusted model^1^		
**Cervical HIV-1 viral load**	**n obs**.	**(N indiv.)**	**Log**_**10**_ **copies/swab**^2^	**β**	**95% CI**	***P***	**β**	**95% CI**	***P***
No schistosomiasis	317	(117)	2.87	Ref	—	—	Ref	—	—
Schistosomiasis	86	(35)	2.70	-0.12	-0.49, 0.25	0.522	0.07	-0.20, 0.34	0.623
**Vaginal HIV-1 viral load**	**n obs**.	**(N indiv.)**	**Log**_**10**_ **copies/swab**^2^	**β**	**95% CI**	***P***	**β**	**95% CI**	***P***
No schistosomiasis	327	(118)	2.66	Ref	—	—	Ref	—	—
Schistosomiasis	84	(35)	2.62	-0.02	-0.36, 0.31	0.893	0.11	-0.17, 0.39	0.459

^1^ Adjusted for age (16-24/25-34/≥35), year of HIV-1 acquisition (4-year bands), and log_10_ plasma viral loads.

^2^ The mean log_10_ copies/swab genital viral loads for individuals with and without schistosomiasis, it does not take into account multiple observations per individual.

Since ova from *S*. *haematobium* more frequently induce genital damage than ova from *S*. *mansoni*, potentially leading to inflammation and elevated genital viral load levels, we performed subgroup analyses for each schistosome species (Table 4). After adjusting for age, year of HIV-1 acquisition, and plasma viral load levels, *S*. *mansoni* infection was not associated with a statistically significant change in cervical (0.29 log_10_ copies/swab, 95% CI -0.04 to 0.62) or vaginal (0.16 log_10_ copies/swab, 95% CI -0.19 to 0.52) set point viral loads. In adjusted models, *S*. *haematobium* infected individuals had lower set point cervical viral loads (-0.59 log_10_ copies/swab, 95% CI -1.11 to -0.06) but similar set point vaginal viral loads (-0.09 log_10_ copies/swab, 95% CI -0.65 to 0.46) compared to schistosome-uninfected individuals.

**Table 4:** Associations between schistosome species and HIV-1 genital viral loads in the Mombasa Cohort.

			Bivariate				Adjusted model^1^		
**Cervical HIV-1 viral load**^2^	**n obs**.	**(N indiv.)**	**Log**_**10**_ **copies/swab**^3^	**β**	**95% CI**	***P***	**β**	**95% CI**	***P***
No schistosomiasis	317	(117)	2.87	Ref	—	—	Ref	—	—
*S*. *mansoni*	54	(21)	2.72	0.01	-0.47, 0.46	0.983	0.29	-0.04, 0.62	0.089
*S*. *haematobium*	18	(8)	2.54	-0.31	-1.04, 0.42	0.404	-0.59	-1.11, -0.06	0.029
Undetermined species^4^	20	(8)	2.80	-0.02	-0.73, 0.70	0.965	0.11	-0.40, 0.62	0.672
**Vaginal HIV-1 viral load**^2^	**n obs**.	**(N indiv.)**	**Log**_**10**_ **copies/swab**^3^	**β**	**95% CI**	***P***	**β**	**95% CI**	***P***
No schistosomiasis	327	(118)	2.66	Ref	—	—	Ref	—	—
*S*. *mansoni*	52	(21)	2.58	-0.08	-0.50, 0.34	0.707	0.16	-0.19, 0.52	0.367
*S*. *haematobium*	18	(8)	2.83	0.09	-0.56, 0.74	0.718	-0.09	-0.65, 0.46	0.738
Undetermined species^4^	20	(8)	2.73	0.16	-0.47, 0.80	0.615	0.18	-0.36, 0.72	0.507

^1^ Adjusted for age (16-24/25-34/≥35), year of HIV-1 acquisition (4-year bands), and log_10_ plasma viral loads.

^2^ In both analyses there were 3 individuals (7 observations) who tested positive for both *S*. *mansoni* and *S*. *haematobium*.

^3^ The mean log_10_ copies/swab genital viral loads for individuals with and without schistosomiasis, it does not take into account multiple observations per individual.

^4^ These individuals had antischistosomal antibodies (anti-SEA) and schistosome antigens (CAA >10 pg/ml), but tested negative for both *S*. *mansoni* and *S*. *haematobium*.

## Discussion

Using data from 370 individuals who HIV-1 seroconverted during prospective follow-up and who contributed 1,407 plasma and 814 genital samples, we found no evidence supporting our hypothesis that schistosomiasis increases HIV-1 plasma or genital viral loads. In fact, *S*. *mansoni* infection was associated with a statistically significant decline in plasma viral loads, while *S*. *haematobium* infection was associated with a statistically significant decline in cervical viral loads.

Our findings conflict with a recent study in Tanzania that found *S*. *mansoni* associated with increased HIV-1 set point plasma viral loads [9]. Interestingly, HIV-1 viral load levels in the two studies were similar among individuals with *S*. *mansoni* (4.2 vs. 4.4 log_10_ copies/ml) but schistosome-uninfected individuals had lower viral load levels in the Tanzania study (4.5 vs. 3.7 log_10_ copies/ml). Though set point plasma viral loads vary by population, in other reports from sub-Saharan Africa they typically range from 4.2 to 4.8 log_10_ copies/ml [22, 38–40]. Thus, the Tanzania study had unusually low plasma viral load levels in their uninfected population, perhaps explained by ART use, which was not monitored in their study population.

Overall, studies that evaluated associations between schistosomiasis and plasma viral load levels in cross-sectional analyses or assessed changes in viral load levels after schistosomiasis treatment have found inconsistent results [8, 9, 41–45]. Since higher set point plasma viral loads are predictive of faster HIV-1 disease progression and mortality [34, 46–50], a positive association between schistosomiasis and plasma viral loads would suggest that schistosomiasis plays a role in catalyzing the HIV-1 epidemic. Results presented in this manuscript represent the largest evaluation of this association done to date, and our results do not support this hypothesis. The schistosomiasis prevalence among our study population was similar to the national prevalence in Kenya (23%) and Uganda (20%) [51], suggesting that our findings may be generalizable to the general populations of these countries. In addition, the fact that high intensity schistosome infections were associated with lower, rather than higher, plasma viral load levels makes it unlikely that we failed to identify a positive association because our study population did not have a sufficient burden of schistosomiasis. Additionally, our results are consistent with a recent study that found schistosomiasis associated with slower, rather than faster, rates of HIV-1 disease progression [52].

Though a positive association between schistosomiasis and genital HIV-1 levels has been hypothesized by others [15, 19, 20], to the best of our knowledge this is the first study to evaluate this association. Since *S*. *haematobium* more frequently causes genital damage than *S*. *mansoni* [23, 24], and the cervix is the genital organ most often damaged by schistosome ova [23], we expected to see the highest genital viral loads among cervical samples collected from women infected by S. *haematobium*. Yet, cervical HIV-1 viral loads were lower in this population. We lack a possible biological mechanism to explain this result and acknowledge that we have only eight women infected by *S*. *haematobium* included in the analysis. This result may have occurred by chance, as we performed multiple statistical tests, increasing the probability of identifying a false association.

Utilizing data from four cohort studies, one strength of our analysis is the large sample size of HIV-1 seroconverters included in the plasma set point analysis. Since these individuals were enrolled in ongoing cohort studies, we were able to estimate their date of HIV-1 acquisition with good precision and utilize data collected on potential confounders such as ART use to address possible sources of bias. Our schistosomiasis testing algorithm allowed us to determine the schistosome species causing the infection, enabling us to perform subanalyses by species. Lastly, the availability of both cervical and genital HIV-1 viral load measurements from the Mombasa Cohort allowed us to assess the impact of schistosomiasis on two female genital organs known to experience damage from schistosome ova.

One limitation of our analysis is that schistosomiasis status was measured at a single time point prior to HIV-1 seroconversion. Thus, it is possible that participants’ schistosome infection status changed during the set point period. We expect most individuals’ schistosomiasis status remained unchanged over the two year period since schistosomiasis acquisition most frequently occurs during childhood [53], individuals in these cohorts were not systematically screened or treated for schistosomiasis, and untreated infections have been shown to persist for up to 40 years [53]. Additionally, to address this concern, we performed a sensitivity analysis restricting sample inclusion to 4–12 months after HIV-1 acquisition, yet we still detected no positive associations between schistosomiasis and HIV-1 set point plasma or genital viral loads.

An additional limitation is that the SCAA20 assay lacks the sensitivity to detect very low-burden infections [30]. This could be a modest source of misclassification, but we would expect low burden infections to induce the least amount of genital damage. Additionally, the fact that our study population included 68 individuals with “undetermined species” of schistosome infection likely indicates that the Western blots used to differentiate between *S*. *mansoni* and *S*. *haematobium* infection were not as sensitive as we anticipated, since existing epidemiological data indicates that no other schistosome species are found in the study area [3]. Since these assays have a specificity of >99% [33], we do not believe that this biased our species-specific results, but instead that it reduced our statistical power in the subanalyses by species by reducing the number of individuals we could classify as *S*. *mansoni* or *S*. *haematobium* infected. Our limited statistical power may have prevented us from detecting more modest associations between schistosomiasis and HIV, and our analysis evaluating associations between *S*. *haematobium* and genital viral loads would have been strengthened by a larger sample size that would have produced more precise point estimates.

In conclusion, we found no evidence that schistosomiasis is associated with increased plasma HIV-1 set point viral loads. Thus, our results do not support the hypothesis that schistosomiasis increases the rate of HIV-1 disease progression. Additionally, we found no evidence that schistosomiasis is associated with increased female genital HIV-1 set point viral loads, providing no evidence to support the hypothesis that schistosome coinfection increases HIV-1 infectivity in women. Though we did not find that schistosomiasis increased plasma or genital HIV-1 viral loads, schistosomiasis induces significant morbidity and mortality, and ongoing support for preventive treatment and schistosomiasis control is needed in many regions of sub-Saharan Africa [54].

## Supporting information

S1 Table Associations between schistosomiasis infection intensity and HIV-1 set point plasma viral load. (DOCX)

S2 Table Associations between schistosomiasis and HIV-1 set point plasma viral load by sex and study population. (DOCX)

S1 Checklist STROBE Checklist. (DOC)
