# Supplementary material for: Schistosomiasis was not associated with higher HIV-1 plasma or genital set point viral loads among HIV seroconverters from four cohort studies
Source: PLoS Negl Trop Dis. 2019 Nov 20;13(11):e0007886. doi: 10.1371/journal.pntd.0007886 (PMC6867600; doi:10.1371/journal.pntd.0007886)
Supplement: S1 Table — (DOCX) [file pntd.0007886.s001.docx]

**S1 Table** Associations between schistosomiasis infection intensity and HIV-1 set point plasma viral load

|  |  |  | **Bivariate** | | | |  | **Adjusted model^1^** | | | |
| --- | --- | --- | --- | --- | --- | --- | --- | --- | --- | --- | --- |
| **All participants** | **n obs.** | **(N indiv.)** | **Log_10_ copies/ml^2^** | **β** | **95% CI** | ***P*** |  | | **β** | **95% CI** | ***P*** |
| No schistosomiasis^3^ | 1102 | (288) | 4.50 | Ref | — | — |  | | Ref | — | — |
| Low intensity | 78 | (21) | 4.32 | -0.05 | -0.42, 0.32 | 0.793 |  | | -0.12 | -0.49, 0.24 | 0.508 |
| Moderate intensity | 113 | (32) | 4.48 | 0.05 | -0.25, 0.36 | 0.731 |  | | 0.04 | -0.26, 0.34 | 0.801 |
| High intensity | 114 | (29) | 4.17 | -0.41 | -0.73, -0.10 | 0.010 |  | | -0.44 | -0.75, -0.13 | 0.006 |

^1^ Adjusted for age (16-24/25-34/≥35), sex, and cohort, plus year of HIV-1 acquisition for the Mombasa Cohort (4-year bands).

^2^ The mean log_10_ copies/ml plasma viral loads for individuals with and without schistosomiasis, it does not take into account multiple observations per individual.

^3^ Definition of infection intensity categories: No schistosomiasis (anti-SEA negative or CAA <10 pg/ml), low intensity (anti-SEA positive & CAA 10-99 pg/ml), medium intensity (anti-SEA positive & CAA 100-999 pg/ml), and high intensity (anti-SEA positive & CAA ≥1000 pg/ml).
