# Supplementary material for: Schistosomiasis was not associated with higher HIV-1 plasma or genital set point viral loads among HIV seroconverters from four cohort studies
Source: PLoS Negl Trop Dis. 2019 Nov 20;13(11):e0007886. doi: 10.1371/journal.pntd.0007886 (PMC6867600; doi:10.1371/journal.pntd.0007886)
Supplement: S2 Table — (DOCX) [file pntd.0007886.s002.docx]

**S2 Table** Associations between schistosomiasis and HIV-1 set point plasma viral load by sex and study population

|  |  |  | **Bivariate** | | | |  | **Adjusted model^1^** | | | |
| --- | --- | --- | --- | --- | --- | --- | --- | --- | --- | --- | --- |
| **All participants** | **n obs.** | **(N indiv.)** | **Log_10_ copies/ml^2^** | **β** | **95% CI** | ***P*** |  | | **β** | **95% CI** | ***P*** |
| No schistosomiasis | 1102 | (288) | 4.50 | Ref | — | — |  | | Ref | — | — |
| Schistosomiasis^3^ | 305 | (82) | 4.32 | -0.14 | -0.34, 0.07 | 0.184 |  | | -0.17 | -0.38, 0.03 | 0.094 |
| **Males, serodiscordant** | **n obs.^4^** | **(N indiv.)** | **Log_10_ copies/ml^2^** | **β** | **95% CI** | ***P*** |  | | **β** | **95% CI** | ***P*** |
| No schistosomiasis | 290 | (62) | 4.40 | Ref | — | — |  | | Ref | — | — |
| Schistosomiasis^3^ | 81 | (21) | 4.37 | 0.05 | -0.37, 0.47 | 0.810 |  | | 0.04 | -0.38, 0.46 | 0.859 |
| **Females, all** | **n obs.^4^** | **(N indiv.)** | **Log_10_ copies/ml^2^** | **β** | **95% CI** | ***P*** |  | | **β** | **95% CI** | ***P*** |
| No schistosomiasis | 812 | (226) | 4.53 | Ref | — | — |  | | Ref | — | — |
| Schistosomiasis^3^ | 224 | (61) | 4.31 | -0.20 | -0.43, 0.04 | 0.097 |  | | -0.23 | -0.46, 0.00 | 0.050 |
| **Females, serodiscordant** | **n obs.^4^** | **(N indiv.)** | **Log_10_ copies/ml^2^** | **β** | **95% CI** | ***P*** |  | | **β** | **95% CI** | ***P*** |
| No schistosomiasis | 303 | (70) | 4.24 | Ref | — | — |  | | Ref | — | — |
| Schistosomiasis^3^ | 69 | (15) | 4.21 | -0.11 | -0.60, 0.38 | 0.654 |  | | -0.09 | -0.58, 0.39 | 0.707 |
| **Females, FSW** | **n obs.^4^** | **(N indiv.)** | **Log_10_ copies/ml^2^** | **β** | **95% CI** | ***P*** |  | | **β** | **95% CI** | ***P*** |
| No schistosomiasis | 509 | (156) | 4.71 | Ref | — | — |  | | Ref | — | — |
| Schistosomiasis^3^ | 155 | (46) | 4.35 | -0.26 | -0.52, -0.00 | 0.049 |  | | -0.29 | -0.55, -0.02 | 0.032 |

^1^ Adjusted for age (16-24/25-34/≥35), sex, and cohort, plus year of HIV-1 acquisition for the Mombasa Cohort (4-year bands). Sub-analyses by sex do not adjust for sex.

^2^ The mean log_10_ copies/ml plasma viral loads for individuals with and without schistosomiasis, it does not take into account multiple observations per individual.

^3^ Individuals with antischistosomal antibodies (anti-SEA) and schistosome antigens (CAA >10 pg/ml).
